# UHPLC-DAD/ESI-TOF-MS Phytochemical Characterization and Evaluation of the Impact of *Eleutherococcus senticosus* Fruit Intractum on Biochemical, Hepatological, and Blood Parameters in Balb/c Mice

**DOI:** 10.3390/ijms25179295

**Published:** 2024-08-27

**Authors:** Filip Graczyk, Jakub Gębalski, Dorota Sulejczak, Milena Małkowska, Magdalena Wójciak, Dorota Gawenda-Kempczyńska, Elżbieta Piskorska, Krystian Krolik, Maciej Markiewicz, Aneta Kondrzycka-Dąda, Wiktoria Lepianka, Grzegorz Borowski, Marcin Feldo, Robert Verporte, Daniel Załuski

**Affiliations:** 1 Department of Pharmaceutical Botany and Pharmacognosy, Ludwik Rydygier Collegium Medicum, Nicolaus Copernicus University, 9 Marie Curie-Skłodowska Street, 85-094 Bydgoszcz, Poland; jakubgebalski@gmail.com (J.G.); milena.malkowska@cm.umk.pl (M.M.); dgawenda@cm.umk.pl (D.G.-K.); krystian.krolik99@wp.pl (K.K.); daniel.zaluski@cm.umk.pl (D.Z.); 2 Department of Experimental Pharmacology, Mossakowski Medical Research Institute, Polish Academy of Sciences, Pawinskiego 5 St., 02-106 Warsaw, Poland; dsulejczak@imdik.pan.pl; 3 Department of Analytical Chemistry, Medical University of Lublin, 4a Chodzki Str., 20-093 Lublin, Poland; magdalena.wojciak@umlub.pl; 4 Department of Pathobiochemistry and Clinical Chemistry, Ludwik Rydygier Collegium Medicum, Nicolaus Copernicus University, 9 Marie Curie-Skłodowska Street, 85-094 Bydgoszcz, Poland; piskorska_e@cm.umk.pl; 5 Department of Soil Science and Landscape Management, Nicolaus Copernicus University, Lwowska 1, 87-100 Toruń, Poland; mawicz@umk.pl; 6 Faculty of Medicine, University of Social Sciences, 9 Sienkiewicza Str., 90-113 Łódź, Poland; akondrzycka-dada@san.edu.pl; 7 J. Brudziński’s Provincial Hospital for Children, Chodkiewicza 44, 85-667 Bydgoszcz, Poland; wiktoria-l@wp.pl; 8 Department of Vascular Surgery and Angiology, Medical University of Lublin, 20-081 Lublin, Poland; grzegorzborowski@umlub.pl (G.B.); martinf@interia.pl (M.F.); 9 Natural Products Laboratory, Institute of Biology, Leiden University, 2300 RA Leiden, The Netherlands; verpoort@chem.leidenuniv.nl

**Keywords:** *Eleutherococcus senticosus*, hepatotoxicity, intractum, *Balb/c* mice, triterpenic acids

## Abstract

*Eleutherococcus senticosus* (Rupr. et Maxim.) Maxim. (ES) has gained popularity for its adaptogenic, immunostimulant, and anti-inflammatory properties. Because of overexploitation of the roots, the species is considered to be endangered and has been put on the Red List in some countries (e.g., the Republic of Korea). Therefore, the fruits of *E. senticosus* might be explored as a new sustainable source of compounds with adaptogenic activity. This study aimed to assess the chemical composition and the safety profile (hepatotoxicity, blood morphology, biochemical parameters of blood plasma) of *E. senticosus* fruit intractum in Balb/c mice after oral administration of 750 and 1500 mg/kg b.w. UHPLC analysis coupled with DAD and MS detectors was used to quantify the metabolites. For the first time, oleanolic and ursolic acids were quantified in the intractum (16.01 ± 1.3 and 2.21 ± 0.17 µg/g of oleanolic and ursolic acids, respectively). Regarding polyphenols, chlorogenic acid (0.92 mg/g of dried extract), caffeic acid (0.43 mg/g), dicaffeoylquinic acids (in total: 1.27 mg/g), and an unidentified caffeic acid ester (0.81 mg/g) were identified. The results in Balb/c mice revealed that the intractum does not cause significant variations in red blood cells parameters. In turn, a significant decrease in the total number of leukocytes was observed (5.8 × 10^3^ µL), with a percentage increase in lymphocytes among the groups (80.2, 81.8, and 82.6). The ability of the intractum to decrease alanine aminotransferase (ALT) and aspartate aminotransferase (AST) levels may indicate its anti-inflammatory activity. Our observations justify that the fruits of *E. senticosus* are safe in the doses used and do not cause significant changes in the activity of the liver enzymes or in blood parameters.

## 1. Introduction

In the ethnomedicine of Russia and China, *Eleutherococcus senticosus* (Rupr. et Maxim.) Maxim. roots, in different pharmaceutical forms, are used to treat immune-related diseases (Table 1). In the 1970s, the Russian researchers began studying this species, focusing on its adaptogenic, immune-boosting, and anti-inflammatory properties. Nowadays, *E. senticosus* is gaining popularity in Europe as a dietary supplement to cure impotence, weakness, and immune system diseases. These indications have been officially accepted in the Community Herbal Monograph on *E. senticosus* (Rupr. et Maxim.) Maxim. Radix (EMEA/HMPC/244569/2006), published by the European Medicines Agency [1,2,3].

**Figure 1:**
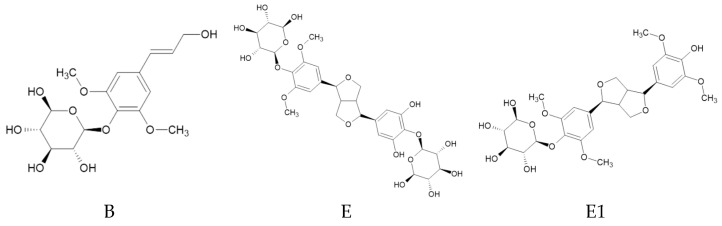
The chemical structures of eleutheroside B (syringin 4-*β*-D-glucoside), eleutheroside E ((−)-siringaresinol 4,4”-*O*-*β*-D-diglucoside), and eleutheroside E1 ((−)-siringaresinol 4-*O*-*β*-D-glucoside).

*E. senticosus* is rich in diverse chemical compounds such as flavonoids, phenolic acids, lignans, saponins, and essential oils. Its main metabolites include eleutherosides, including eleutheroside B, eleutheroside E, and eleutheroside E1, which are mainly responsible for its pharmacological activity (Figure 1). These substances are found in both the aerial and underground parts of the plant. Moreover, *E. senticosus* fruits contain large amounts of polyphenols, flavonoids, and minerals like iron, zinc, and manganese [4,5,6,7].

Furthermore, compounds present in both the fruits and roots of *E. senticosus* have demonstrated the ability to regulate enzyme activity, such as hyaluronidase, indicating its potential in treating conditions through its anti-inflammatory effects [8]. *E. senticosus* is also known to exhibit antiviral activity against respiratory syncytial virus (RSV) and influenza A virus in cell cultures infected with these viruses [9]. *E. senticosus* is also able to reduce the cardiovascular responses to stress, proving to be helpful for stress adaptation [10]. A recent report [11] showed that *E. senticosus* demonstrated significant neuroprotective activity, including regulation of neurotransmitters, inhibition of neuronal cell apoptosis, and mitigation of neuritis. ES leaf extracts also show antidiabetic effects and improve fat metabolism, which can be used to support the treatment of diabetes [12].

Despite promising discoveries, further research on the biochemical and cellular properties of *E. senticosus* is needed to fully understand its mechanisms of action and therapeutic potential.

Over time, *E. senticosus* has been acknowledged as an adaptogen and classified within this category. Most adaptogens originate from the roots of five- and six-year-old plants, which increases the regeneration period. Due to excessive exploitation of the roots, some countries consider it a protected species [13]. Modern approaches advocate utilizing aerial parts like flowers or fruits for medicinal purposes while protecting the roots and rhizomes to avoid endangering the species’ survival. It should be mentioned *E. senticosus* is one of the most important non-timber forest products in northeastern China. According to the latest research results, human activities and climate change have resulted in serious threats to the habitats of the tree. It is suggested that the area of suitable habitat for *E. senticosus* will shrink by the 2090s, so we need to urgently control logging intensity and use the aerial parts of the species [14].

Taking these arguments into consideration, we decided to evaluate the quality of *E. senticosus* fruit intractum prepared from fruits grown in Poland. Our previous experiments proved that *E. senticosus* fruit intractum caused an increase in PBL proliferation and a decrease in viral replication in a VSV–PBL–intractum model. The latest results from Graczyk et al. indicate hyperpolarization reactions in the rabbit large intestine and inhibition of serum hyaluronidase isolated from children diagnosed with acute leukemia [2,15].

Despite extensive research into the phytochemical and phytopharmacological properties of ES, there is a lack of data regarding its phytochemicals and the impact of the fruits on blood parameters and liver activity, as well as their toxicity. We hypothesized that the fruit’s intractum does not contain compounds that may cause changes in the liver and blood after oral administration. To prove this, two doses, i.e., 750 and 1500 mg/kg b.w., were orally administered.

## 2. Results

### 2.1. Phytochemical Composition of the Intractum

**Figure 2:**
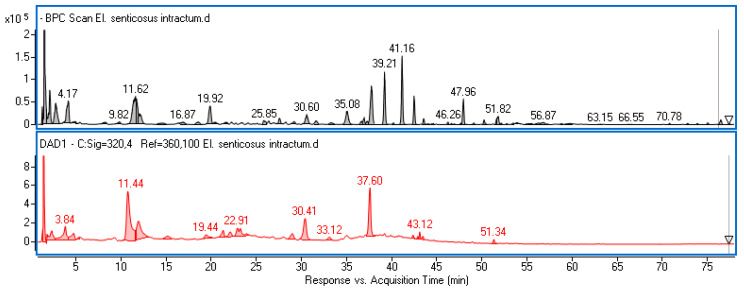
Base peak chromatogram (grey line) and the chromatogram registered at λ = 320 nm (red line) of *E. senticosus* fruit intractum.

Biological activity of extracts is linked with the presence of secondary metabolites, very often characteristic of only a small group of plants. The components of the *E. senticosus* fruit intractum were investigated using ultra-high performance liquid chromatography with DAD and MS detection. An example chromatogram is shown in Figure 2.

Identification of the compounds was carried out based on MS spectra acquired in negative ion mode (*m/z*-H) and DAD spectra in the range of 200 to 400 nm. Chromatographic and spectral data were compared with those obtained for standards and with literature data (Table 2).

The UHPLC analysis revealed the presence of some polyphenolic components in the intractum, with caffeic acid derivatives being predominant. These include chlorogenic acid (0.92 mg/g of dried extract), caffeic acid (0.43 mg/g), dicaffeoylquinic acids (in total: 1.27 mg/g), and an unidentified caffeic acid ester (0.81 mg/g). All these components display a common ion with *m/z*-H = 179 and maximum absorbance in the range of 322 to 327 nm, which is characteristic of caffeic acid derivatives. Furthermore, protocatechuic acid and quinic acid (cyclohexanecarboxylic acid) were found in the intractum in amounts of 0.39 mg/g and 3.51 mg/g, respectively. A flavonoid compound, namely quercetin 3-O-glucoside, was also detected.

Additionally, on the base peak chromatogram, some peaks were observed at retention times ranging from 39 to 52 min. These unidentified components may belong to the sterol or fatty acid classes. They are characterized by a strong MS signal (i.e., they are easily ionizable) and possess a nonspecific UV-Vis spectrum, with maximum absorption at 200–210 nm or no absorption, which indicates a small number of or lack of chromophores.

Analysis of the content of eleutheroside E (di-β-D-glucoside of (−)-syringaresinol) and eleutheroside B (syringin), specific components of *E. senticosus*, shows the presence of only eleutheroside E, with an amount of 0.96 mg/g. The detailed results of the qualitative and quantitative analyses of the polyphenolic constituents of the intractum are summarized in Table 3.

### 2.2. Soil Properties

The results are presented in Table 4. The soil in the garden was formed as a result of spreading peat-like material on a loamy substrate. Five years ago, the soil was fertilized with granulated cattle manure. The structure of the soil material is very small granules and single grains, and quite numerous small shells are visible. The texture is loamy sand. Currently, in terms of properties, the surface horizon is consisting of material that fulfills the criteria for *mulmic* material, in accordance with WRB classification [16], or *arenimurshic* material, according to Polish soil classification [17]. *Mulmic* material (from the German Mulm, meaning powdery detritus) is mineral material developed from organic material after dehydration. This process led to intensive mineralization and a decrease of organic matter content. Currently, the soil contains over 8% of organic carbon, and the C:N ratio is 16. Such a value is the optimum ratio for soil microbes to stimulate release of nutrients like N, phosphorous, and zinc for crops [18,19]. On the other hand, the soil is alkaline (pH 7.4), which may result in lower availability of most nutrients for plants [20]. Calcium (Ca^2+^) dominates in the soil sorption complex. The use of Mg-containing fertilizers, e.g., kiesierite, would be advised for improvement of the Ca:Mg ratio in the sorption complex. Although this study did not conduct additional analyses to evaluate the impact of soil on the metabolite content in *E. senticosus* extract or on the studied parameters in mice, soil studies provide information on the soil conditions under which E. senticosus was cultivated. This is particularly important because *E. senticosus* is a geographically non-native species in Poland and is found only under cultivation. Moreover, it should be noted that environmental factors, including soil parameters, have a crucial impact on the growth and development of plants, thereby also influencing the content of active compounds in medicinal plant materials. The level of metabolite content, in turn, affects the therapeutic efficacy of the raw materials [21,22].

The parameters presented represent the mean values calculated for each group of mice (*n* = 5), plus the standard deviation.

### 2.3. Body Weight and Organs Parameters

**Figure 3:**
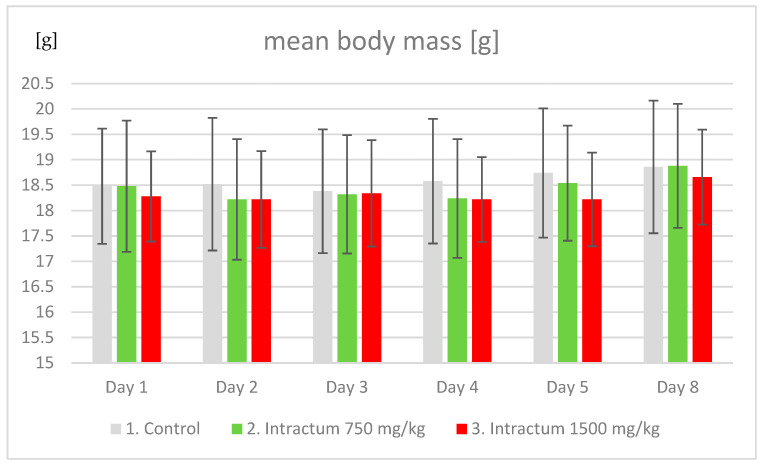
The change in average body weight (g) in groups of mice treated with different doses of intractum (mg/kg b.w.).

Throughout the experiment, mouse body weight was measured daily. The average weight of each mouse in the groups increased with time, which is consistent with expectations. In the case of the third group, which was stimulated with the highest dose of the intractum, the weight gain was significantly lower than that of the control group (Figure 3). However, this difference was not statistically significant.

**Figure 4:**
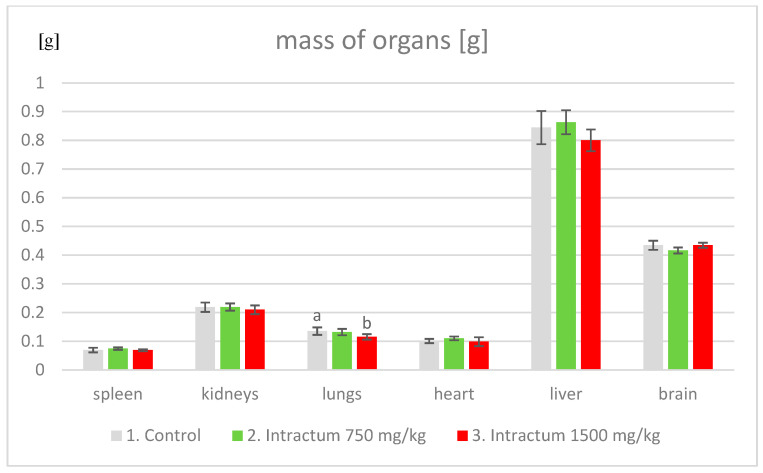
Average weight of individual organs collected from groups of mice; results presented as mean ± SD. Different letters (a, b) indicate statistically significant differences, with *p* < 0.05.

After the mice were euthanized, the livers, spleens, kidneys, lungs, hearts, and brains were collected and weighed (Figure 4). In the case of the tested group of mice taking two doses of intractum, no statistically significant differences were observed. However, statistically significant differences were observed exclusively in the lung mass of the control group and the group administered 1500 mg/kg of intractum.

### 2.4. Blood Parameters

The parameters of red blood cells were examined, which are summarized in the Table 5. No significant changes or deviations from were found in any of the studied group.

Next, the white blood cells were examined (Table 6). The studies showed that, in the group of mice treated with intractum (the dose of 1500 mg/kg b.w.), a significant decrease in the total number of leukocytes was observed (5.8 × 10^3^ µL), with a percentage increase in lymphocytes among the groups (80.2, 81.8, and 82.6).

The next part of the blood tests concerned biochemical parameters. The results for each group of mice presented in the Table 7 showed that there were no statistically significant differences in alanine aminotransferase (ALT) and aspartate aminotransferase (AST) activity between the control and intractum 750 mg/kg b.w. groups; however, there was a significant decrease in ALT and AST levels in the intractum 1500 mg/kg b.w. group: ALT 27, 27, and 22.2 and AST 113.6, 109.3, and 104.1 U/L. There was no difference in creatinine level among the groups (12 µmol/L), a slight decrease in urea level (6.3–5.7 mmol/L, *p* = 0.03) and total protein (44.8–42 g/L, *p* = 0.045) was observed for the group treated with the dosage of the intractum at 1500 mg/kg b.w. Statistical analyses showed a statistically significantly higher content of urea and total protein in the control group compared to the group treated with the dosage of the intractum at 750 mg/kg b.w.

### 2.5. Histopathological Analyses

Histopathological evaluation did not reveal any pathological changes in the livers obtained from either the control animals or those obtained from the animals treated with the various concentrations of the intractum. The cells showed proper morphology and architecture, and the livers had a normal tissue structure.

**Figure 5:**
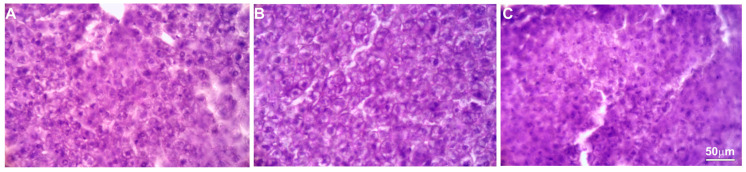
H&E staining. Microphotographs of representative liver sections obtained from control mice (**A**) and animals treated with 750 (**B**) or 1500 mg/kg b.w. (**C**) of the intractum.

Only in two of the five mice tested were single areas of brighter H and E labelling observed, but the cells in these areas showed no signs of degeneration and had proper morphology and arrangement (Figure 5).

### 2.6. Statistics

**Figure 6:**
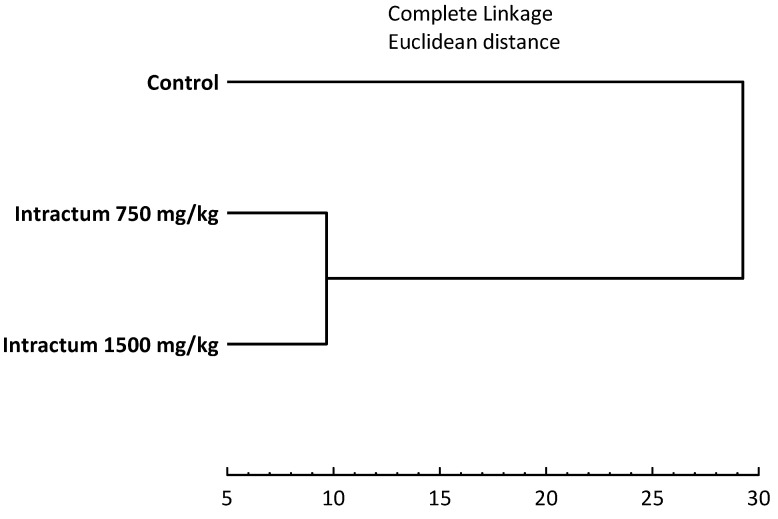
Hierarchical cluster analysis of three analyzed groups of mice based on all examined parameters.

Hierarchical cluster analysis (Figure 6) indicates that there are similarities in the health profile of mice administered intractum at doses of 750 mg/kg and 1500 mg/kg. Both the 750 mg/kg and 1500 mg/kg doses caused comparable effects in the studied parameters. Simultaneously, the dendrogram shows a distinct difference between the parameters of mice given intractum and the control group.

**Figure 7:**
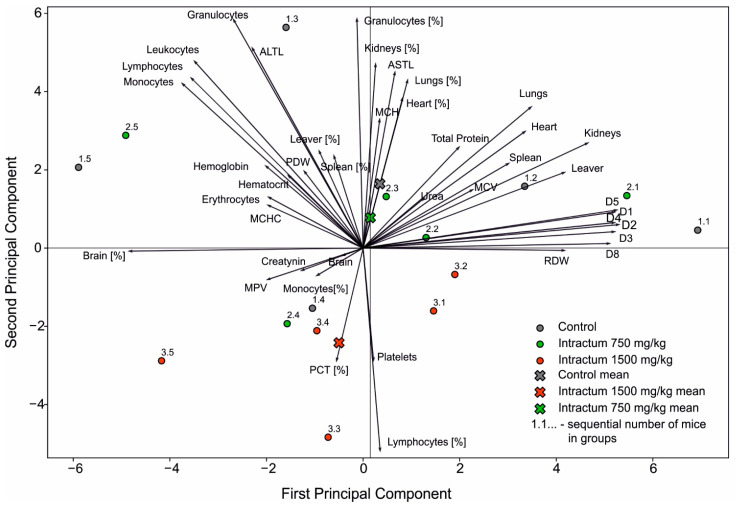
Principal component analysis of analyzed groups of mice based on all examined parameters.

Principal component analysis (Figure 7) based on all studied parameters indicates a separation of the mean values of the three studied groups of mice. However, the parameters for individual specimens from the group administered intractum at 750 mg/kg and mice from the control group overlap. This may suggest that the 750 mg/kg dose does not introduce significant changes in the profile of the studied parameters compared to the control group. The group of mice given intractum at 1500 mg/kg appears to be more separated from the others, especially from the control group, indicating that intractum administration at this concentration has a greater impact on the tested parameters than the 750 mg/kg concentration.

The location of the point representing the mean values for the group of mice administered intractum at 1500 mg/kg suggests a positive correlation with platelet count and PCT and a negative correlation with, among others, the quantity of granulocytes, ASTL, MCH, and the percentage size of the lungs, heart, and kidneys. The first principal component accounted for 29.35% of the variance, while the second principal component explained an additional 15.61%.

Based on the extracted ion chromatogram (EIC) mode, ursolic acid (*m/z*-H 455.35307, estimated formula C_30_H_48_O_3_), belonging to the triterpene class, along with a low amount of its isomer oleanolic acid, were also detected in the sample. Their amounts were 16.01 ± 1.3 µg/g and 2.21 ± 0.17 µg/g, respectively.

## 3. Discussion

In our study, we examined the influence of oral administration of *E. senticosus* fruit intractum (750 and 1500 mg/kg b.w.) on the blood and liver parameters of healthy mice. *E. senticosus* extracts, obtained from the roots and fruits, have been extensively studied for their immunostimulatory and anti-hyaluronidase activity. However, there is no comprehensive information in the literature regarding the effect of *E. senticosus* fruit intractum on changes in weight gain or the effect of short-term supplementation with high doses on individual blood parameters, as well as on the activity of liver cells.

The results obtained clearly showed that the high dose of intractum used did not have a negative impact on the activity of liver cells. The intractum did not exhibit any hepatotoxic activity on liver parenchyma cells, taking into account both the results of blood parameters and histopathological examination. However, high doses of the extract administered to mice induced some changes in blood plasma parameters.

Administration of both the 750 mg/kg b.w. and 1500 mg/kg b.w. doses of intractum for an 8-day cycle resulted in mice gaining less weight compared to the control group. Body weight gain was the lowest at the highest dose, but the differences did not show statistical significance.

Despite the lack of data in this field of research, there are reports that support the validity of the conducted research. It is a well-known fact that metabolism in adipocytes and muscle cells influences changes in overall body mass. In [23] the authors provided information that ES ethanolic root extract (1.0 mg/mL ES) has the potential to promote fat loss and enhance the metabolic profiles of muscles by elevating the expression of proteins associated with lipolysis and lipid metabolism in 3T3-L1 adipocytes and C2C12 skeletal muscle cells. Their results indicated that ES extracts, particularly at relatively higher concentrations (e.g., 1.0 mg/mL ES), effectively reduce triglyceride (TG) content. This effect is likely attributed to the upregulation of lipases (e.g., ATGL) in adipocytes and the increased expression of metabolism-associated proteins (e.g., AMPK, ACC), along with proteins related to mitochondrial biogenesis (e.g., VDAC) in muscle cells. The authors of [24] proved with a rat model that the administration of stem bark *E. senticosus* extract (100 mg/kg b.w.) protects bone mass, reducing its loss in osteoporosis. The extract contributed to maintaining proper bone mass and also reduced AST concentration, which is likewise consistent with our analyses.

Regarding histopathological effects on the liver, applying various doses of *E. senticosus* intractum confirmed our assumption that it does not cause a hepatotoxic effect, as we did not observe any liver damage. This confirms reports about the safety of using ES in ethnomedicine. Additionally, [25] reported that the combination of metformin treatment with the administration of ES water extract (200 mg/kg b.w.) contributed to relieving diabetes symptoms and reversing liver and kidney damage to normal levels compared to administering metformin alone in diabetic rats, with along with improvement of blood parameters and body weight. ES extracts not only do not have a negative effect on hepatocytes, but can also protect the liver against deposition of heavy metals such as cadmium (administration of 3.5 mL/L ES stem water extract) [26].

Moreover, [27] demonstrated results that water extract derived from the root bark of *E. senticosus* (500 or 1000 mg/kg b. w.) enhances fatty acid *β*-oxidation in skeletal muscle, with no impact on body or adipose tissue weights. Additionally, it expedited recovery from physical fatigue induced by forced swimming, which is in line with our observations about the impact of the intractum on organ mass and overall body weight.

Adaptogenic plants should not have a negative impact on the body, which results from the definition of an adaptogen as a plant supporting the abilities of the body to overcome illnesses. Therefore, adaptogens should not negatively affect blood parameters, such as by reducing white blood cell count or changing the percentage profile of the white blood cell content. It was observed that the administration of 1500 mg/kg b.w. had a statistically significant effect on the number of white blood cells, causing their decrease, while the lymphocyte fraction was increased. This effect was not observed in the group fed with a dose of 750 mg/kg body weight, which suggests that supplementation with this dose is safe and preferred for the body, without causing any effect involving white blood cells, such as inflammation. High doses of ES extracts can increase WBCs; indeed, [28] discovered in a group of 36 healthy patients that supplementation with an ethanolic solution of the *Eleutherococcus senticosus* root extract (2 g/day) increased lymphocyte numbers, especially T-helper cells. Maintaining the right amount of WBCs is beneficial in the fight against cancer, for example, which was confirmed by [29], who proved the beneficial effect of the use of ES extract on WBCs during anticancer therapy. The authors discovered, while examining a group of 107 patients with gastric cancer, that daily supplementation with ES root extracts contributed to better tolerance of chemotherapy and influenced the increase in WBCs. Moreover, they proved that patients had a longer survival rate after gastrectomy (from 12 to 17 months).

Regarding its influence on blood parameters, the intractum may indirectly affect biochemical parameters, such as ALT and AST levels, but also total protein, creatine, and urea levels, which may be due to its beneficial effects on stress, inflammation, and overall health. For example, by reducing stress levels, ES intractum may help improve kidney function and influence creatine and urea levels in the blood. Our research showed that administration of both lower and higher doses of the drug contributed to a significant reduction in the level of urea in the blood. These reports confirm those previously provided by [30], who found that ethanol–water solutions of ES root extract (500 mg/kg b.w.) have a positive effect on reducing blood urea nitrogen. This is in line with our observations; although there were differences in the chemical composition of the root extract and the intractum, both exhibited a similar effect.

Similarly, by exerting anti-inflammatory effects, the intractum may help modulate liver activities. ALT and AST are enzymes found primarily in hepatocytes. They play essential roles in various metabolic processes within the liver. Both enzymes are commonly measured as part of liver function tests to assess liver health and function. Lower levels of ALT and AST in the blood are generally considered normal and may indicate healthy liver function [31,32]. Our results show that supplementation with ES intractum in both doses causes the levels of these enzymes to decrease, as well as the levels of total protein in the blood. This could imply that, in addition to having a beneficial effect on the general condition of hepatocytes, the intractum potentially has a positive effect on their metabolic processes, such as influencing total protein levels. Our analyses confirm those given by [33], who observed in a 24-week experimental model that involved 24-week-old Sprague–Dawley male rats fed with the dried product of ethanol ES root and stem extracts (7 mg/day) a significant reduction of all liver-related parameters with long-term supplementation. In [34], the authors also reported similar results using a mouse model (male C57BL/6 mice), proving that intraperitoneal injection with aqueous ES root extracts (300 mg/kg b.w.) or oral administration with the same extract caused notably reduced serum levels of tumor necrosis factor-α, aspartate transaminase, and alanine transaminase. Additionally, it enhanced histologic liver changes and suppressed hepatocyte apoptosis, as confirmed by the study. Similar reports are given by other researchers, who have proven that ethanolic fruit extract from another adaptogenic plant species, *Schisandra chinensis,* decreased AST and ALT levels in Sprague–Dawley rat blood after 14-day pretreatment of rats with a high dose of the extract (1200 mg/kg b.w.) [35].

The results obtained are in agreement with the previous Graczyk et al. results, including immunostimulatory and anti-inflammatory activity. The results indicated hyperpolarization reactions in the rabbit large intestine and inhibition of serum hyaluronidase isolated from children diagnosed with acute leukemia [2,15].

Chemical compounds are responsible for the biological activity of plants. The chemical profile of the intractum is very well known and has been already published by Graczyk et al. We detected in the intractum, for the first time, oleanolic and ursolic acids [16.01 ± 1.3 and 2.21 ± 0.17 µg/g, respectively]. What is interesting is that the intractum, on the basis of the previous results, does not contain eleutherosides, which are generally recognized as adaptogenic and immunostimulative compounds [2,8]. However, using a mass detector, our present results have revealed a small amount of eleutheroside E, i.e., 0.96 ± 0.07, although no eleutheroside B was detected. This amount, probably, is insufficient to be pharmacologically active. In this place it should be mentioned eleutherosides are present in the *E. senticosus* fruits, but not in the fruit intractum, which is prepared from the fresh fruits with 40% ethanol. On the basis of the results obtained in this article and those published by Graczyk et al. [2], we can suggest that derivatives of caffeic acid and *myo*-inositol are responsible for the activity of the intractum. These compounds are known for their immunostimulant and anti-inflammatory activities.

## 4. Materials and Methods

### 4.1. Plant Material

The mature fruits of *Eleutherococcus senticosus* (Rupr. et Maxim.) Maxim. were collected at the Garden of Medicinal and Cosmetic Plants in Bydgoszcz, Poland, in September 2022 (coordinates: 53°07′36.55′′ N, 18°01′51.64′′ E) and stored at the Department of Pharmaceutical Botany and Pharmacognosy, Collegium Medicum, Bydgoszcz, Poland. ES fruit intractum was prepared following the procedure previously outlined by [8]. To verify the identity of the plant materials, a combination of morphological examination and the HPLC-DAD and HPLC-RID analyses were conducted, comparing the results with reference data.

Briefly, fresh fruits (20 g) were macerated in 100 mL of 40% ethanol for 30 days at room temperature, kept away from light. Next, the extract was filtered using Whatman no. 4 filter paper. The solvent was removed by evaporation at 45 °C, then frozen at −20 °C and subjected to lyophilization [2]. The extraction resulted in 6.1% dry extract. The dried residue was stored in an exicator at 4 °C and labeled as “intractum” for further analysis.

### 4.2. Chromatographic Analysis

The extract was separated using an ultra-high performance liquid chromatograph (UHPLC) Infinity Series II with a DAD detector and an Agilent 6224 ESI/TOF mass detector (Agilent Technologies, Santa Clara, CA, USA). The conditions were as follows: an RP18 reverse-phase column Titan (Supelco, Sigma-Aldrich, Burlington, MA, USA) (10 cm × 2.1 mm i.d., 1.9 µm particle size), a thermostat temperature of 30 °C, and a flow rate of 0.2 mL/min. Water with 0.1% formic acid (solvent A) and acetonitrile with 0.1% formic acid (solvent B) were used as the mobile phase. The gradient elution program was as follows: 0–12 min 95% A, 12–20 min from 95% A to 85% A, 20–30 min 85% A, 30–50 min from 85% A to 50% A, 50–60 min 50% A, and 60–85 min from 50% A to 0% A. LC–MS conditions: drying gas temperature 325 °C, drying gas flow 9 L min^−1^, nebulizer pressure 30 psi, capillary voltage 3500 V, and skimmer 65 V. The voltage on the fragmentator was 220 V. Ions were acquired in the range of 100 to 1200 *m/z* in negative ion mode. All standards, formic acid, and MS-grade acetonitrile were from Sigma-Aldrich (St. Louis, MO, USA).

Identification was performed based on literature data and by comparing the retention times, fragmentation patterns, and UV-Vis spectra with standards when they were available. The other components were tentatively identified based on UV-Vis spectra and mass data. Formulas were calculated using MassHunter software (Version 3.3.2 SP2 build 3.3.2.1037). Quantification was performed using an external calibration method. The calibration curves were constructed by plotting peak areas versus standard concentrations. Solutions and extracts were analyzed in triplicate. For each point, the relative standard deviation did not exceed 3.2%. The coefficient of determination (r^2^) for each calibration curve was higher than 0.99. The calibration equations were as follows: y = 8454x + 6.1 (λ = 254 nm) for eleutheroside E, y = 6250x + 12.5 (λ = 254 nm) for protocatechuic acid, y = 7038x − 0.46 (λ = 325 nm) for caffeic acid, y = 9410x − 5.92 (λ = 325 nm) for chlorogenic acid, y = 8407x + 0.93 (λ = 254 nm) for quercetin 3-O-glucoside, y = 7038x − 0.46 (λ = 325 nm) for 3,5-dicaffeoylquinic acid, y = 6687x − 0.85 (λ = 325 nm) for 4,5-dicaffeoylquinic acid, y = 24 158 359x + 13 745 (EIC) for quinic acid, and y = 115 109 093x − 697 (EIC) for ursolic acid.

### 4.3. Soil Analysis

In the laboratory, the garden soil sample was air-dried, disaggregated, homogenized, and sieved through a 2 mm sieve. The following soil properties were determined in the prepared sample: CaCO_3_ content using the Scheibler volumetric method; pH at a soil-to-solution ratio of 1:2.5 using 1 M KCl and distilled H_2_O as the suspension medium; content of total carbon (TC) and total nitrogen (TN) determined by dry combustion (Vario MacroCube, Elementar, Langenselbold, Germany), following which the TC content was corrected with content of inorganic carbon to determine the total organic carbon content (TOC); exchangeable base cation (Ca^2+^, Mg^2+^, K^+^, Na^+^) content after extraction with 1 M ammonium acetate at pH 8.2, measured by a SOLAR 969 atomic absorption spectrophotometer (Unicam, Cambridge, UK).

### 4.4. Animals and Experiments

A group of female Balb/c mice, aged 7 weeks, was housed in a pathogen-free environment. The mice were maintained under the conditions of a 12/12-h day/night cycle with unrestricted access to food and drinking water. These mice were randomly allocated into four groups and accommodated in microisolator cages equipped with sterilized feed and autoclaved bedding. After a week of acclimatization, three groups of mice (*n* = 5) were subjected to treatment for five consecutive days: one group received water (group I), while the other two groups were administered varying doses of the intractum—750 mg/kg (group II) and 1500 mg/kg (group III) based on body weight.

Throughout the treatment period, the mice were weighed daily. On the eighth day all mice were humanely sacrificed under deep anesthesia (Morbital, 150 mg/kg). Blood samples were collected and centrifuged at 2000× *g* at 4 °C for 15 min, and the resulting sera were preserved at −80 °C for subsequent analysis. The activity of ALT and AST was assayed. Additionally, the levels of creatinine, urea, and total protein, as well as blood morphology, were measured.

Following euthanasia, the liver, spleen, kidneys, lungs, and brain were promptly removed, weighed, and fixed in 4% formaldehyde in 0.1 M phosphate buffer (pH 7.4) for 2 days, and next were processed according to standard procedures for microscopic examination.

### 4.5. Blood Morphological and Biochemical Analyses

Whole blood was collected in a tube containing low-molecular-weight heparin (LMWH) at 5000 IU/mL and then analyzed using a Mythic 18 hematology analyzer (C2 Diagnostics, Montpellier, France). Then, the blood specimens were centrifuged at 2000× g for 15 min at 4 °C. After this step, the plasma was transferred to fresh tubes and frozen at −20 °C for further biochemical standard analysis.

Alanine aminotransferase (ALT), aspartate aminotransferase (AST), urea, creatinine, and total protein were measured in plasma samples in a Cobas C 111 analyzer (Roche Diagnostics, Rotkreuz, Switzerland) using reagents and procedures provided by the manufacturer.

### 4.6. Histopathological Analyses

To determine hepatocyte morphology, the livers were assessed histopathologically. Fixed livers were cryopreserved by immersion in 10, 20, and 30% (*w/v*) sucrose solutions in PBS (for 24 h, 3 days, and 6 days, respectively). In the next step, the livers were frozen on dry ice, cut into 20 µm thick sections using a CM 1850 UV cryostat (Leica, Wetzlar, Germany), and mounted on glass. Sections were subjected to morphological studies using routine hematoxylin and eosin (H&E; hematoxylin No. 1.50174, and eosin No. 1.09844, Merck Millipore, Warsaw, Poland) staining. Next, the stained sections were cover-slipped with DPX (slide mounting medium, Sigma-Aldrich, Schenlldorf, Germany) and analyzed and captured using a Nikon light microscope (Nikon, Tokyo, Japan) equipped with a CCD camera ((Nikon, Tokyo, Japan) and image analysis system.

Approval for the animal experiments was obtained from the Local Committee for Ethical Animal Experiments of the Hirszfeld Institute of Immunology and Experimental Therapy, Poland, with the assigned approval number 058/2022/P1.

### 4.7. Statistics

Statistically significant differences were checked using one-way ANOVA, for which assumptions of normality and homogeneity of variances were verified and met. The Tukey test was used as a post hoc test. Hierarchical cluster analysis (HCA) and principal component analysis (PCA) were utilized to identify differences among the three studied mouse groups, employing Statistica 12 software for statistical computations.

## 5. Conclusions

To sum up, our research justifies the traditional use of *Eleutherococcus senticosus* fruit and intractum, proving supplementation to be rather safe. These data are novel, as this was one of the first well-controlled study of ES intractum supplementation and demonstration of its non-hepatotoxic effects. Considering the obtained results, a dosage of 750 mg/kg b.w. or lower is recommended for future research involving an extended administration period of 4 weeks. However, it is clear that a dose of 1500 mg/kg b.w. could have a potential short-term application in the case of increased ALT and AST levels. The ability to decrease ALT and AST levels may indicate an anti-inflammatory effect, which is consistent with the previous report by Graczyk et al. [15]. The fruits exhibit potential as a sustainable, plant-based adaptogen, making the species potentially suitable for cultivation in Western Europe, especially in Poland. Nevertheless, additional in vivo studies focusing on bioavailability are needed to fulfill all the EU criteria concerning plant-based drugs and medicines utilized in traditional healing systems. Given its rich biological properties and extensive use in Asian cultures, there is potential for it to gain popularity in European countries, but further investigations are needed.

## Figures and Tables

**Table 1:** Summary of the pharmaceutical forms and dosages for oral administration of *Eleutherococcus senticosus* preparations [2].

Pharmaceutical Form	Roots				Fruits
	Powder	Tincture (1:5)	Fluid Extract (1:1)	Solid Extract (20:1)	Intractum
Doses	2–4 grams/1–3 divided doses daily	10–20 mL/1–3 divided doses daily	2–4 mL/1–3 divided doses daily	100–200 mg/1–3 divided doses daily	No data available
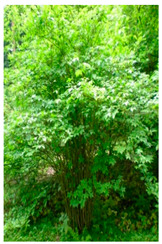	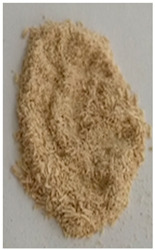	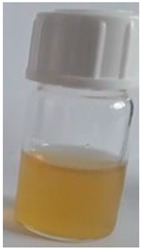	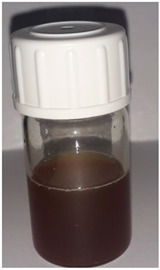	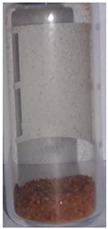	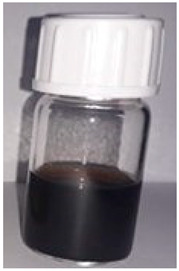

**Table 2:** MS spectra of phenolic components identified in intractum from the fruits of *Eleutherococcus senticosus* and standards used for their identification.

Sample	Standard
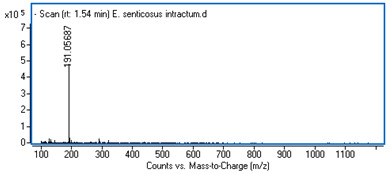	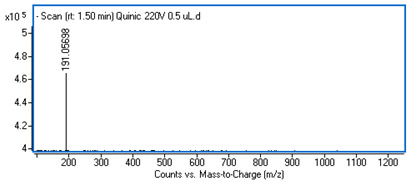
Quinic acid	
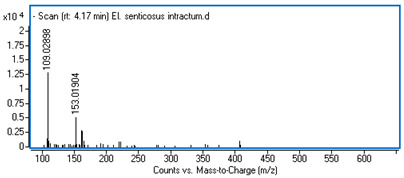	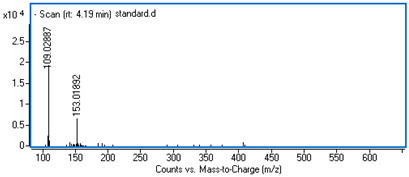
Protocatechuic acid	
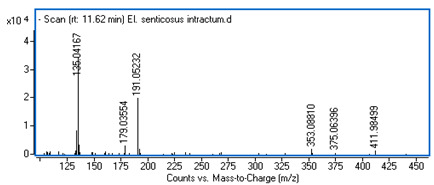	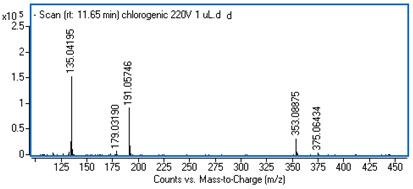
Chlorogenic acid	
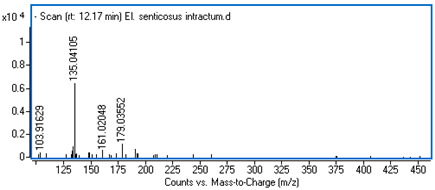	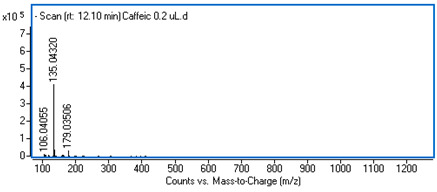
Caffeic acid	
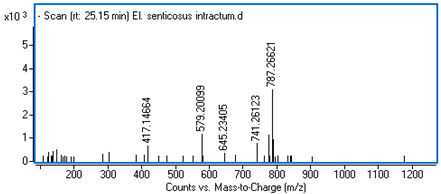	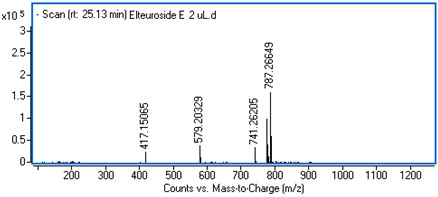
Elteutheroside E	
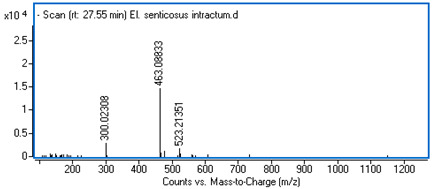	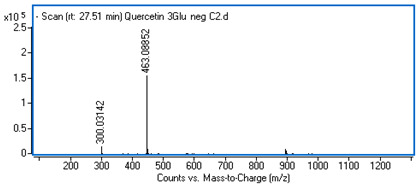
Quercetin 3-O-glucoside	
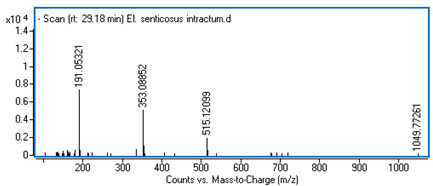	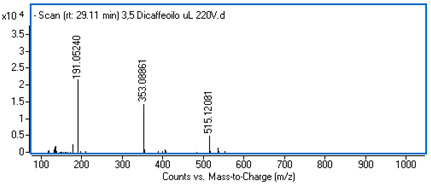
3,5-dicaffeoylquinic acid	
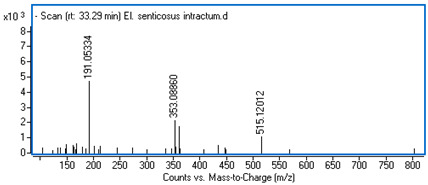	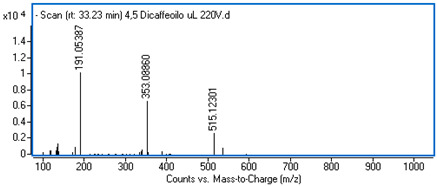
4,5-dicaffeoylquinic acid	

**Table 3:** MS data and the results of quantifying phenolic components (mg/g ± SD) identified in extract from the fruit of *E. senticosus*.

Rt (min)	Observed Ion Mass [M-H]-/(Fragments)	Δ ppm	Formula	Identified	Content (mg/g)
1.53	191.05687	3.95	C_7_H_12_O_6_	Quinic acid	3.51 ± 0.22
4.17	153.01904	−1.90	C_7_H_6_O_4_	Protocatechuic acid	0.39 ± 0.02
11.62	353.0881 (135,179,191)	0.83	C_16_H_18_O_9_	Chlorogenic acid	0.92 ± 0.07
12.17	179.03552 (135)	2.99	C_9_H_8_O_4_	Caffeic acid	0.43 ± 0.02
25.15	741.26123 (417, 579, 787 *m/z* +HCOOH)	0.12	C_34_H_46_O_18_	Eleutheroside E	0.96 ± 0.07
27.55	463.08833 (300)	0.28	C_21_H_20_O_12_	Quercetin 3-O-glucoside	0.18 ± 0.02
29.18	515.12099 (191,353)	2.89	C_25_H_24_O_12_	3,5-dicaffeoylquinic acid	0.34 ± 0.02
30.60	515.12048 (191,353)	1.90	C_25_H_24_O_12_	Dicaffeoylquinic acid	0.72 ± 0.05
33.29	515.12012 (191,353)	1.20	C_25_H_24_O_12_	4,5-dicaffeoylquinic acid	0.21 ± 0.01
37.74	207.06673 (179)	2.15	C_11_H_12_O_4_	Caffeic acid ester *	0.81 ± 0.06

* Quantification was based on the calibration curve for caffeic acid.

**Table 4:** Selected soil properties.

Soil Sample	pH		CaCO_3_	TOC	TN	C:N	Ca^2+^	Mg^2+^	K^+^	Na^+^	CEC
	H_2_O	KCl	%				cmol(+)kg^−1^				
1	7.40	7.40	9.53	8.60	0.537	16	18.44	0.07	0.23	0.12	18.87

**Table 5:** Selected red blood cell parameters for the control group and the groups treated with 750 or 1500 mg/kg b.w.

Parameter	Control	Intractum 750 mg/kg	Intractum 1500 mg/kg
Hemoglobin [g/dL]	15.3 ± 0.9	15.1 ± 1.2	15.3 ± 1.0
RBCs [10^6^/µL]	8.6 ± 0.5	8.5 ± 0.7	8.7 ± 0.6
Hematocrit [%]	37.0 ± 1.8	36.1 ± 3.0	36.9 ± 2.2
MCV [fL]	43.2 ± 1.1	42.2 ± 0.3	42.4 ± 0.5
MCH [pg]	17.9 ± 0.2	17.7 ± 0.4	17.6 ± 0.1
MCHC [g/dL]	41.4 ± 1.0	41.8 ± 0.7	42.6 ± 0.5
RDW [%]	18.3 ± 1.2	18.0 ± 0.2	17.9 ± 0.4
PLT [10^3^/µL]	470.4 ± 62.7	499.0 ± 85.2	504.4 ± 82.9
MPV [fL]	5.9 ± 0.5	6.2 ± 0.3	5.9 ± 0.3
PDW [fL]	40.8 ± 4.4	40.3 ± 2.0	42.3 ± 2.0
PCT [%]	0.278 ± 0.035	0.310 ± 0.061	0.299 ± 0.050

**Table 6:** Selected white blood cell parameters for the control group and the groups treated with 750 or 1500 mg/kg b.w.

Parameter	Control	Intractum 750 mg/kg	Intractum 1500 mg/kg
WBCs [10^3^/µL]	7.9 ± 2.1	7.8 ± 2.0	5.8 ± 0.9
Lymphocytes [10^3^/µL]	6.3 ± 1.6	6.4 ± 1.6	4.8 ± 0.8
Monocytes [10^3^/µL]	0.4 ± 0.1	0.4 ± 0.1	0.3 ± 0.1
Granulocytes [10^3^/µL]	1.2 ± 0.4	1.0 ± 0.4	0.7 ± 0.2
Lymphocytes [%]	80.2 ± 2.8	81.8 ± 2.7	82.6 ± 1.7
Monocytes [%]	5.0 ± 0.05	5.0 ± 0.6	5.2 ± 0.8
Granulocytes [%]	14.8 ± 2.4	13.2 ± 2.4	12.2 ± 1.4

**Table 7:** The chosen blood parameters for the control group and the groups treated with 750 or 1500 mg/kg b.w. Different letters (a, b) in the rows indicate statistically significant differences, with *p* < 0.05.

Parameter	Control	Intractum 750 mg/kg	Intractum 1500 mg/kg
ALT [U/L]	27.0 ± 6.2	27.0 ± 6.3	22.2 ± 3.2
AST [U/L]	113.6 ± 23.4	109.3 ± 22.5	104.1 ± 13.1
Creatinine [µmol/L]	12.2 ± 1.7	12.8 ± 1.7	12.0 ± 1.1
Urea [mmol/L]	6.3 ± 0.7 ^a^	5.3 ± 0.4 ^b^	5.7 ± 0.5
Total protein [g/L]	44.8 ± 2.2 ^a^	41.6 ± 1.3 ^b^	42.0 ± 1.9

## Data Availability

The raw data supporting the conclusions of this article will be made available by the authors on request.
